# Intraganglionic reactive oxygen species mediate inflammatory pain and hyperalgesia through TRPA1 in the rat

**DOI:** 10.3389/fpain.2023.1204057

**Published:** 2023-05-30

**Authors:** Youping Zhang, Jamila Asgar, Huizhong Shou, Joshua Pak, Joyce Teixeira Da Silva, Jin Y. Ro

**Affiliations:** Department of Neural and Pain Sciences, University of Maryland School of Dentistry, Baltimore, MD, United States

**Keywords:** sensory ganglia, muscle pain, inflammation, oxidative stress, antioxidant, hyperalgesia

## Abstract

Reactive oxygen species (ROS) are generated in nociceptive pathways in response to inflammation and injury. ROS are accumulated within the sensory ganglia following peripheral inflammation, but the functional role of intraganlionic ROS in inflammatory pain is not clearly understood. The aims of this study were to investigate whether peripheral inflammation leads to prolonged ROS accumulation within the trigeminal ganglia (TG), whether intraganglionic ROS mediate pain hypersensitivity via activation of TRPA1, and whether TRPA1 expression is upregulated in TG during inflammatory conditions by ROS. We demonstrated that peripheral inflammation causes excess ROS production within TG during the period when inflammatory mechanical hyperalgesia is most prominent. Additionally, scavenging intraganglionic ROS attenuated inflammatory mechanical hyperalgesia and a pharmacological blockade of TRPA1 localized within TG also mitigated inflammatory mechanical hyperalgesia. Interestingly, exogenous administration of ROS into TG elicited mechanical hyperalgesia and spontaneous pain-like responses via TRPA1, and intraganglionic ROS induced TRPA1 upregulation in TG. These results collectively suggest that ROS accumulation in TG during peripheral inflammation contributes to pain and hyperalgesia in a TRPA1 dependent manner, and that ROS further exacerbate pathological pain responses by upregulating TRPA1 expression. Therefore, any conditions that exacerbate ROS accumulation within somatic sensory ganglia can aggravate pain responses and treatments reducing ganglionic ROS may help alleviate inflammatory pain.

## 1. Introduction

Contributing to a wide range of physiological processes, reactive oxygen species (ROS) are highly reactive derivatives of molecular oxygen produced as a byproduct of normal enzymatic reactions (1). When redox homeostasis is dysregulated, excess ROS accumulate in cells and leads to oxidative stress. A pathological increase in ROS levels can directly damage DNA, protein, and lipids, and initiates cascades of cellular and molecular events that culminate into the development of various pathological conditions, including chronic pain (2, 3).

ROS contribute to a variety of pain conditions at multiple levels of nociceptive pathways, including inflammatory pain, neuropathic pain, cancer pain, and chemotherapy-induced pain, among others. For example, tissue injury or inflammation leads to increased ROS production in local tissues (4, 5, 6, 7) and scavenging of local ROS prevents peripheral sensitization (7, 8, 9). ROS in local tissues also promote peripheral sensitization through their reciprocal interaction with TRPV1 (10), by increasing endogenous agonists for TRPA1 via lipid peroxidation (11, 12, 13) and by modulating the expression of proinflammatory cytokines and chemokines (6, 14, 15).

ROS generated in the spinal cord following nerve injury can lead to central sensitization of spinal cord dorsal horn neurons via phosphorylation of N-methyl-D-aspartate (NMDA) receptors (16), phosphorylation and cell surface localization of *α*-amino-3-hydroxy-5-methyl-4-isoxazolepropionic acid (AMPA) receptors (17), and through a loss of gamma-aminobutyric acid (GABA) neurons and dysfunction of surviving GABA neurons (18). In addition, ROS enhances excitatory synaptic transmission in rat spinal cord dorsal horn neurons by activating TRPA1 and TRPV1 at the central terminals of primary afferent neurons (19). ROS levels are also elevated within trigeminal ganglia (TG) of rats following peripheral inflammation (20). However, there is limited information on the functional role of intraganglionic ROS under pathological pain conditions.

It is well established that TRPA1 functions as a detector of both environmental irritants and endogenous mediators generated during injury or inflammation (21), and that TRPA1 in both peripheral and central terminals of nociceptive afferents instigates pathological pain conditions (22). Moreover, the expression level of TRPA1 correlates with mechanical hypersensitivity in several inflammatory pain models (23, 24, 25). Interestingly, ROS can directly activate TRPA1, which is also a redox sensitive channel (11). ROS can also induce TRPA1 gene expression via the generation of inflammatory cytokines (26, 27). Therefore, it is possible that ROS generated in the immediate vicinity of primary afferent cell bodies can directly modulate TRPA1 function and expression.

The purpose of this research was to examine whether inflammation in the masseter muscle causes a prolonged accumulation of ROS in TG. Additionally, the study sought to determine if intraganglionic ROS plays a role in pain hypersensitivity by activating TRPA1 and increasing its expression in the TG during inflammation. Understanding these questions is important because intraganglionic ROS can generate and sustain pain responses, even when there is no accumulation of ROS in the peripheral or central sites. Moreover, reducing ROS in various parts along the nociceptive pathway could result in more effective pain relief. Furthermore, it is significant to comprehend the functional role of TRPA1 within sensory ganglia, as TRPA1 activation in these ganglia can independently contribute to pain responses.

## 2. Materials and methods

### 2.1. Animals

Adult male Sprague-Dawley rats (3 months old; 150 to 350 g; Harlan, IN, USA) were used. All animals were housed in a temperature-controlled room under a 12:12 light-dark cycle with access to food and water *ad libitum*. All procedures were conducted in accordance with the National Institutes of Health Guide for the Care and Use of Laboratory Animals (publication no. 80–23) and under a University of Maryland Baltimore approved Institutional Animal Care and Use Committee protocol.

### 2.2. Masseter inflammation

Inflammation was induced by injecting 50 μl of 50% Complete Freund's Adjuvant (CFA) in isotonic saline (Sigma-Alridch, St. Louis, MO) into the mid-region of the masseter muscle via a 27-gauge needle. Rats were briefly anesthetized with 3% isoflurane for the injection procedure. The characteristics of inflammation following CFA injections in the rat masseter have been described previously (28, 29).

### 2.3. ROS assay in TG

The methods for the ROS assay were described in our previous study (20). Briefly, ROS levels were quantified using a cell-permeant oxidant-sensing probe 2′,7′-dichlorodihydrofluorescein diacetate (H_2_DCFDA; Invitrogen, Carlsbad, CA, USA). H_2_DCFDA is de-esterified within the cytoplasm and turns highly fluorescent upon oxidation. H_2_DCFDA detects hydrogen peroxide (H_2_O_2_), peroxyl radicals (ROO•), and peroxynitrite (ONOO−), but it is possible that other biologically relevant ROS, such as superoxide radicals (O2•−) and hydroxyl radicals (OH•), are also involved. Rats were injected with either CFA or the same volume of vehicle into the left masseter muscle. Naïve rats that did not receive either CFA or vehicle treatment served as a control group. The TG ipsilateral to the injected muscle was removed either 1, 4, 7, 14, or 28 days after the injection. TG was quickly removed and washed with phosphate-buffered saline (PBS). Immediately after extraction and dissection, the tissues were minced finely in PBS and were incubated in 96-well plates in 200 *μ*l PBS for 30 min at 37°C. The background fluorescence for each specimen was determined with a fluorimeter (DTX880 Multimode Detector, Beckman Coulter) at 485 nm for excitation and 535 nm for emission. After the background reading, H_2_DCFDA was added to each well to a final concentration of 10 *μ*M. The plates were again incubated for 30 min at 37°C, and the fluorescence was re-measured. ROS levels were estimated as the intensity of fluorescence after subtraction of the background fluorescence (Multimode Analysis Software). To minimize experimental variations, samples from naïve, CFA-, and vehicle-treated groups at each time point were analyzed on the same day. The results from CFA- or vehicle-treated group were normalized to the results from naïve rats at each time point. We have previously shown that negative control groups without TG tissues (PBS alone, PBS with H_2_DCFDA, PBS with H_2_O_2_, PBS with H_2_O_2_ and H_2_DCFDA) generated little or no positive signal, which is only approximately 1% or less of signals obtained from TG tissues (20). We have also shown that TG samples with exogenously added H_2_O_2_ exhibit robust fluorescence signal in the presence of H_2_DCFDA (20). These control groups validate that our methods allow reliable detection of ROS in TG tissues by H_2_DCFDA.

### 2.4. Microinjections into TG

All microinjections into TG were made through a 26-gauge guide cannula (P1 Technologies, Roanoke, VA, USA) that was surgically implanted over TG (2.5 mm posterior and 1.5 mm lateral to the bregma). Briefly, the scalp was incised 1 cm in length with a sterile scalpel at the midline. A small hole was drilled in the skull using a dental drill with a sterile burr bit (2.35 mm shank, 2.7 × 2.5 mm) to enable implantation of a sterile 26-gauge guide cannula (Roanoke, VA) over TG (2.5 mm posterior and 1.5 mm lateral to the bregma) using stereotaxic coordinates. The cannula was secured to the skull using pharmaceutical grade acrylic cement and two small screws (3 mm) inserted into the parietal bones. The incision edges were cleansed with Betadine and rinsed with 0.9% saline and will be closed with a Wax or Silicone coated braided non-absorbable suture (4-0 or 5-0) material in a simple interrupted pattern. Sutures were removed 7 to 10 days after surgery.

The rats were briefly anesthetized with isoflurane (> 3%–4.5% Induction-chamber) (> 1.5%–3% Maintenance—nosecone) for microinjection procedures. The depth of anesthesia was confirmed by pinching the toe using serrated forceps. All microinjections were made manually using a sterile 30-gauge injection cannula attached to a 1.0 ml syringe (Hamilton, Reno, NV, USA) via PE tubing (0.2 mm ID, 1.75 mm OD). The external end of the cannula was prepped using Betadine solution / scrub and rinsed with alcohol prior to removal of access plug. The substances were infused over a 30 s period and the injection syringe was left in place for an additional 30 s to prevent backflow.

### 2.5. Behavioral studies

#### 2.5.1. Assessment of inflammatory mechanical hyperalgesia

Persistent mechanical hyperalgesia in the masseter muscle was assessed under CFA-induced inflammatory conditions utilizing a behavioral model specifically developed for testing masseter sensitivity in awake rats (30). The rats were habituated to the environment and the experimenter for five consecutive days prior to the behavioral testing. They were trained to stand on a soft pad and lean against the experimenter's hand wearing a leather work-glove, rather than standing on a meshed metal or grid surface. The habituation required no more than gentle petting, and it was achieved within half an hour. In this model, a series of calibrated von Frey filaments (1–125 gm) were applied to the region over the masseter muscle. The rats were not restrained in any way but remained in position long enough for the experimenter to probe the skin overlying the masseter muscle with von Frey filaments.

An active withdrawal of the head from the filament application was defined as a positive response. Each von Frey filament was applied five times and the response frequencies (i.e., (number of responses/ number of stimuli)×100%) to a range of filament forces were determined. After a non-linear regression analysis, an EF50 value, defined as the filament force (g) necessary to produce a 50% response frequency, was determined. The EF50 value was used as a measure of mechanical threshold. A reduction of EF50 after inflammation suggested the presence of mechanical hypersensitivity. The threshold data were subsequently converted to logarithmic values for statistical analyses. Mechanical sensitivity of the masseter muscle was determined prior to and 1, 4, 7, 14, 21 and 28 days after the CFA injection in the masseter muscle.

To examine the role of intraganglionic ROS in inflammatory mechanical hyperalgesia, a ROS scavenger, phenyl N-tert-butylnitrone (PBN) [0.1 mg in 5 *μ*l, a dose shown to produce analgesic effects (9, 31, 32)] or vehicle control (PBS), was administered directly into TG. For preemptive scavenging of ROS, PBN or vehicle was administered 30 min prior to the CFA treatment. For the post-CFA scavenging of ROS, PBN or vehicle was administered 1, 3 and 7 days after CFA treatment in the same animal. The effect of PBN or vehicle on CFA-indued mechanical sensitivities was assessed one hour later. The contribution of intraganlionic TRPA1 in inflammatory mechanical hyperalgesia was examined similarly to the PBN experiment detailed above. AP18 (20 mM in 5 *μ*l), a TRPA1 antagonist, or the same volume of vehicle (1% DMSO, 10% Tween80 in PBS) was administered directly into TG 1, 3 and 7 days after CFA treatment in the same animal. The post AP18 or vehicle effect on masseter mechanical sensitivity was measured 40 min after the injection. The concentration of AP18 we used was higher than what we used in previous behavior studies, where AP18 was administered intramuscularly (33, 34). We chose to use a higher concentration of AP18 because we were making a focal injection into the trigeminal ganglion with a very small volume (5 *μ*l).

#### 2.5.2. Evaluation of spontaneous muscle pain

We used the Rat Grimace Scale (RGS) assay to examine whether exogenous administration of H_2_O_2_ directly into TG can elicit spontaneous pain and whether AP18 treatment can attenuate the H_2_O_2_-induced pain. *Video Imaging*: Rats were acclimated to the testing environment for 2 to 3 days prior to the behavioral assessment. Rats were placed in a transparent, 9 × 5 × 8-inch acrylic container during behavioral experiments. Behavior was monitored via two cameras (Sony Handycam HDR-CX405) placed on the opposing, long sides of the container. For baseline data collected prior to an injection in TG, rats were placed in the container for 5 min of habituation before facial expressions were recorded for 10 min. Following recording the baseline data, H_2_O_2_ (20 *μ*M in 5 *μ*l) was co-administered with AP18 (20 mM in 5 *μ*l) or vehicle (1% DMSO, 10% Tween 80 in PBS) directly into TG. Forty minutes after the intraganlionic administration, changes in facial expressions were recorded for an additional 10 min. *Scoring System for RGS:* To capture face image of rats in an unbiased manner, images were manually extracted from each 10-minute video segment by a blinded observer. When possible, one image was captured at every 60 s interval within the 10-minute recording time. Images were defined as valid and saved for further analysis if the image contained a clear shot of the whiskers, and at least one eye and ear. Images were not captured during sleeping, active sniffing, or grooming. Up to 10 images of each rat were obtained and cropped to enlarge facial features. Video analysis did not always yield 10 valid images, and in these cases the 60 s intervals that did not provide valid images were skipped and no image was captured. Two blinded scorers rated four action units of RGS for each image. Four specific facial action units (AUs) were scored: orbital tightening, nose and cheek flattening, ear position, and whisker change (35, 36). The observer scored 0, 1, or 2 for each AU based on the criteria described previously by Sotocinal et al., (2011) (36). The scores of the four AUs were averaged to yield a RGS score for each individual image. The RGS scores of every image, belonging to one animal, were averaged to yield an overall RGS score for that animal.

### 2.6. Dissociation of rat TG neurons

The procedures for primary TG cultures are described previously (37, 38, 39). Both TG from each animal were dissected out and dissociated by sequential digestion with 0.1% collagenase D in DMEM-F12 medium (with L-glutamine) at 37°C for 30 min, followed by additional digestion in a medium containing 0.25% trypsin, 50 *μ*g DNase, and 0.02% EDTA at 37°C for 15 min. After trituration, cells were plated on laminin pre-coated 24-well plates and cultured in a 37°C incubator at 5% CO_2_ for 1 to 3 days.

### 2.7. Real-time RT-PCR

To examine whether ROS are involved in the transcription of the TRPA1 gene in TG neurons, we took two complimentary approaches. First, we treated TG primary culture with H_2_O_2_ (10 *μ*mol, 1 h). Second, we administered a ROS donor, t-BOOH (2 *μ*mol/10 *μ*l, 2 consecutive days), directly into TG of intact animals without inflammation. Total RNA was extracted from either the dissociated TG cells in culture or dissected TG from intact animals, respectively, using an RNeasy kit (Qiagen Sciences, Germantown, MD) followed by DNase treatment to remove genomic DNA. Reverse transcription was carried out using SuperScript II kit (Invitrogen, Waltham, MA) was used to generate cDNA from 500 ng of RNA along with 2.5 ng of random primer per reaction. Real-time PCR analysis of cDNA (equal to 15 ng of RNA) was performed using Maxima SYBR Green/ROX qPCR Master Mix in an Eppendorf Mastercycler Ep Realplex 2.0 (Fermentas, Forest City, CA, USA). In all our RT-PCR experiments, each sample was analyzed in triplicates, and we routinely added a control with no template as a means of checking for any nucleic acid contamination, and a control with no reverse transcriptase to verify that there was no DNA contamination in the RNA preparation. The no template control also serves to identify any potential formation of primer dimers during the SYBR Green assay. The following primer pairs were used to detect *Trpa1* mRNA: forward 5′-TCCTATACTG GAAGCAGCGA-3′, reverse 5′-CTCCTGATTGCCATC GACT-3′, and GAPDH, mRNA, used as a control: forward 5′-TCACCACCAT GGAGAAGGC G-3′, reverse 5′-GCTAAGCAGTTGGTG GTGCA-3′. We obtained the ratios between *Trpa1* and GAPDH to calculate the relative abundance of mRNA levels in each sample. Relative quantification of the *Trpa1* mRNA was calculated by the comparative CT method (2^−^*^ΔΔ^*^CT^ method) between control and experimental groups.

### 2.8. Western blotting

Total proteins were extracted from the TG of naïve and CFA treated rats. The protein samples were dissolved in RIPA buffer containing protease inhibitor cocktail. The protein concentration of lysates was determined using Bio-Rad protein assay kit (Bio-Rad, Hercules, CA, USA). Fifty micrograms of protein for each sample were separated on 4%–12% NuPAGE gel with MOPS SDS running buffer and transferred to a PVDF membrane (Bio-rad, Hercules, CA, USA). After blocking for 1 h in 5% milk PBST at room temperature, membranes were probed with primary antibodies for TRPA1 (1:5,000, Millipore #ABN-1009, Burlington, MA) and an internal control protein GAPDH (1:5,000, Calbiochem, San Diego, CA), diluted in blocking solution. The TRPA1 antibody was raised against the N-terminus of rat TRPA1 and detects a 90–98-kDa protein, which disappears in TG lysates probed with TRPA1 antibody pre-incubated with a commercially available peptide used to generate the antibody. We have validated the specificity of this antibody in our previous study (33). Membranes from TG samples were incubated with primary antibodies overnight at 4°C and washed four times with PBST. HRP-conjugated secondary antibodies (anti-rabbit secondary antibody (Cell Signaling, Danvers, MA) and anti- mouse secondary antibody (Millipore, Burlington, MA) were diluted to 1:5,000 in PBST and incubated with membranes for 1 h at room temperature. Bands were visualized using ECL (Western Lightning, PerkinElmer Inc., Waltham, MA, USA) or ECL plus Western blotting detection reagent (Lumigen PS-3, GE Healthcare, Chicago, IL). Protein level for TRPA1 was normalized to that of GAPDH within the same sample.

### 2.9. Statistical analyses

The time-dependent changes in mechanical hyperalgesia before and after CFA or vehicle were analyzed with a Two-Way analysis of variance (ANOVA) with repeated measures. Data obtained from RT-PCR and Western blot experiments were analyzed with a one-way ANOVA on means or Kruskal–Wallis one-way ANOVA on ranks depending on the outcome of a normality test. Unless otherwise indicated, statistical comparisons of two independent groups were made with either Student's t- test or Mann–Whitney Rank Sum test. Data are presented as mean ± SE and differences were considered significant at *p* < 0.05. All multiple group comparisons were followed by Bonferroni *post hoc* test. Power analyses were conducted to determine the minimum sample size required for the behavioral experiments to detect a significant effect with a power of 0.8. We used G*Power Software (Heinrich-Heine, Universität Düsseldorf) to confirm that the sample sizes we used yielded a power greater than 0.85 even with a moderate effect size of 0.5.

## 3. Results

### 3.1. CFA treatment in the masseter muscle results in prolonged mechanical hyperalgesia and ROS upregulation within TG

**Figure 1:**
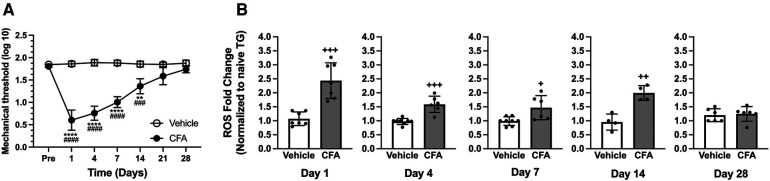
CFA-induced masseter hyperalgesia and ROS upregulation in TG. (**A**) Line graph shows changes in mechanical hyperalgesia in the masseter muscle following CFA (50 *μ*l of 50% CFA in saline) or vehicle (saline) administration (Intramuscular). Mechanical force (g) that produced the head withdrawal responses in 50% of the trials was log transformed and plotted for pre- and 1, 4, 7, 14, 21, and 28 days post CFA treatment. Two-way ANOVA with repeated measures were used. **p < 0.001 and ****p < 0.0001 for significant time effects compared to the pre-injection values. ^###^*p *< 0.001 and ^####^*p *< 0.0001 for significant differences between CFA and vehicle groups (*n* = 8). (**B**) Changes in ROS within TG following masseter inflammation were assessed by measuring relative intensity of fluorescence using H2DCFDA, an indicator for ROS, of TG obtained from naive, CFA, or vehicle treated rats on days 1, 4, 7, 14 and 28 post CFA treatment. We used 5 to 8 naïve rats for normalization for each time point. Student t-test was used for statistical analysis at each time point. ^+^*p *< 0.05, ^++^*p *< 0.005 and ^+++^*p *< 0.0005 for significant differences between CFA and vehicle groups. Data are presented as the mean ± SEM.

We previously demonstrated that an injection of CFA in the rat masseter induces a time-dependent and significant decrease in mechanical thresholds that lasts over 3 weeks (33, 38). We confirmed these results, showing the development of mechanical hypersensitivity following CFA treatment in the masseter. Our data show that there were significant treatment (*F* = 157.7) and time (*F* = 89.20) effects, as well as significant interactions between treatment and time (*F* = 96.31), with a peak decrease in mechanical thresholds during the first 3 days (*p* < 0.0001 for pre-injection vs. post-CFA values, and *p* < 0.0001 for CFA vs. vehicle groups). A significant decrease in mechanical threshold was observed until day 14 (*p* < 0.001 for pre-injection vs. post-CFA values and *p* < 0.0001 for CFA vs. vehicle groups), which gradually returned to a baseline level by 28 days after CFA treatment (Figure 1A). The vehicle treatment did not alter mechanical thresholds for the entire observation period of 28 days (Figure 1A).

Our previous study demonstrated ROS levels increase within TG 1 day after masseter inflammation by CFA (20). A more comprehensive temporal profile of intraganglionic ROS under inflammatory conditions needs to be determined to firmly establish the relationship between inflammatory hyperalgesia with intraganglionic ROS. The inflammation-induced production of ROS in TG was assessed by comparing the intensity of fluorescence from ipsilateral TG obtained 1, 4, 7, 14, and 28 days following the injection of CFA or vehicle into the masseter muscle to that of naïve rats. Fluorescent signals from the TG of CFA-injected rats were significantly greater than those from vehicle-treated rats on days 1, 4, 7, and 14 after CFA treatment (Figure 1B, *p* < 0.0005 for days 1 and 4 post CFA vs. vehicle groups, *p* < 0.05 for day 7 post CFA vs. vehicle groups, and *p* < 0.005 for day 14 post CFA vs. vehicle groups). On day 28, intraganglionic levels of ROS were no longer significantly different between the two groups (Figure 1B). We have previously shown that the ROS level in TG contralateral to the CFA-injection site was not significantly different from that of naïve rats (20). These results demonstrate that masseter inflammation upregulates ROS production within TG, especially during the period when inflammatory mechanical hyperalgesia is pronounced. We conducted the present study using only male rats since the CFA condition we employed produces comparable levels and duration of masseter hyperalgesia in both male and female rats (38). We assumed that shared mechanisms account for the similar behavioral responses in both sexes under our CFA condition. However, we acknowledge that there may be potential differences in ROS accumulation between males and females that require further investigation.

### 3.2. Scavenging of ROS within TG attenuates CFA-induced mechanical hyperalgesia

**Figure 2:**
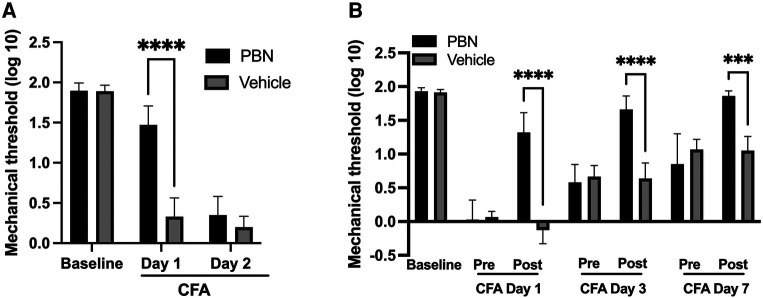
Effects of ROS scavenger (PBN) administration in TG on CFA-induced mechanical hyperalgesia. (**A**) Phenyl N-tert-butylnitrone (PBN—0.1 mg in 5 *μ*l of PBS) or vehicle was injected directly into TG (intraganlionic) 30 min prior to CFA injection. Mechanical hyperalgesia was attenuated significantly in PBN, but not vehicle (PBS), treated rats when examined 1 day after CFA treatment in the masseter. No PBN effects on day 2 post CFA. (**B**) PBN or vehicle was administered directly into TG (intraganlionic) 1 day, 3 days and 7 days after CFA injection in the masseter. Each day, mechanical sensitivity was assessed one hour after PBN or saline administration. Two-way ANOVA with repeated measures were used. *p < 0.01, **p < 0.005 and ***p < 0.0005 for significant differences between PBN and vehicle treated groups. Data are presented as the mean ± SEM.

To examine the functional involvement of intraganglionic ROS in the development of mechanical hyperalgesia, phenyl N-tert-butylnitrone (PBN) was injected directly into TG 30 min prior to CFA treatment in the masseter muscle. There were significant treatment (*F* = 29.33) and time (*F* = 390.3) effects, as well as significant interactions between treatment and time (*F* = 55.98). Preemptive intraganglionic PBN, but not vehicle, treatment significantly attenuated mechanical hyperalgesia one day after CFA treatment (Figure 2A, *p* < 0.005 for PBN vs. vehicle groups). However, PBN was no longer effective on day 2 (Figure 2A). We then examined whether scavenging of ROS within TG can also attenuate fully developed inflammatory mechanical hyperalgesia. There were significant treatment (*F* = 21.24) and time (*F* = 165.0) effects, as well as significant interactions between treatment and time (*F* = 47.09). PBN administered in TG one day after CFA treatment in the masseter muscle significantly blunted mechanical hyperalgesia (Figure 2B, *p* < 0.01 for PBN vs. vehicle groups). The PBN effect was transient, lasting only a few hours. Once the effect wears off, hyperalgesia returned as shown by pre-treatment measures at each time point. Interestingly, when the same concentration of PBN was administered in the same animals again on day 3 after CFA treatment, it blocked the CFA-induced mechanical hyperalgesia to a greater extent (Figure 2B, *p* < 0.0005 for PBN vs. vehicle groups). When PBN was administered a third time on day 7, the mechanical threshold was restored to the pre-CFA baseline sensitivity (Figure 2B). Vehicle administration in TG did not alter mechanical sensitivity at any of the time points (Figure 2B). These data demonstrate that excess production of ROS within TG remote from a peripheral inflammatory site contributes to the development and maintenance of inflammatory hyperalgesia.

### 3.3. TRPA1 within TG mediates CFA-induced mechanical hyperalgesia

**Figure 3:**
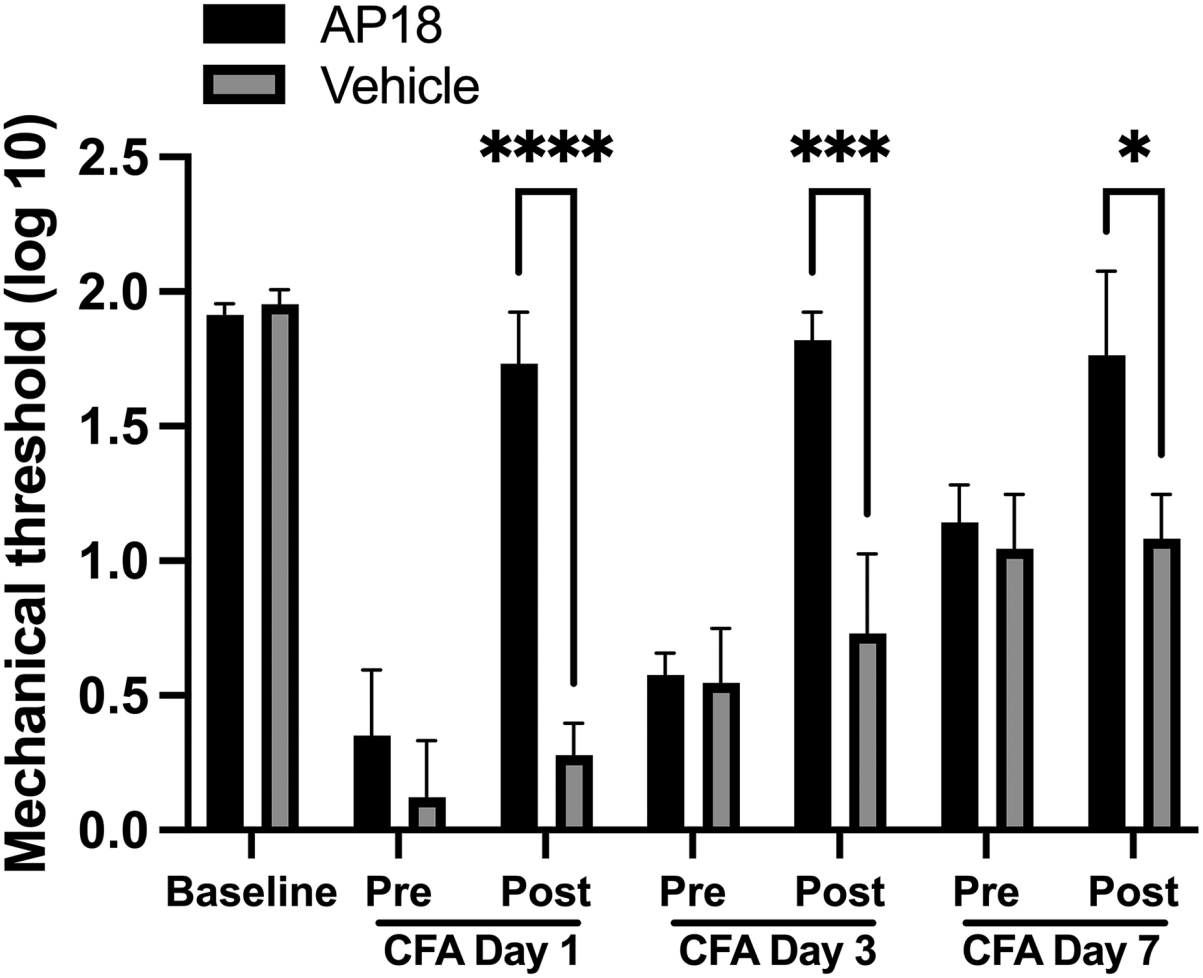
Effects of TRPA1 antagonist (AP18) treatments in TG on CFA-induced mechanical hyperalgesia. AP18 (20 mM in 5 *μ*l) or vehicle (1% DMSO, 10% Tween80 in PBS) was administered directly into TG (intraganlionic) 1 day, 3 days and 7 days after CFA injection in the masseter. Each day, mechanical sensitivity was assessed one hour after AP18 or vehicle administration. Two-way ANOVA with repeated measures were used. *p < 0.005 and **p < 0.0005 for significant differences between PBN and vehicle treated groups. Data are presented as the mean ± SEM.

We have previously shown that CFA-induced mechanical hypersensitivity and spontaneous muscle pain responses were significantly reversed by post-treatment with AP18 in the muscle (33). We have also shown that TRPA1 is amply expressed in the soma of small to medium size TG neurons (34). In order to examine whether TRPA1 expressed within TG could also functionally contribute to inflammatory hyperalgesia, we administered AP18 directly into TG 1, 3 and 7 days after CFA treatment in the masseter muscle. AP18, but not the vehicle, administration in TG one day after CFA treatment in the masseter muscle significantly attenuated the mechanical hyperalgesia (Figure 3, *p* < 0.0005 for AP18 vs. vehicle groups). The inflamed muscle, however, became hypersensitive again within 24 h of AP18 treatment (Figure 3). The same concentration of AP18 administered in the same animals both 1 day and 3 days after CFA treatment blocked the CFA-induced mechanical hyperalgesia to a similar extent of single AP18 treatment on day 1 (Figure 3, *p* < 0.0005 for AP18 vs. vehicle groups). The additional administration of AP18 on day 7 continued to be effective (*p* < 0.005 for AP18 vs. vehicle groups), but it did not further reduce the mechanical hyperalgesia beyond the extent observed on days 1 and 3.

### 3.4. Direct injection of ROS into TG induces mechanical hyperalgesia and facial pain in a TRPA1-dependent manner

**Figure 4:**
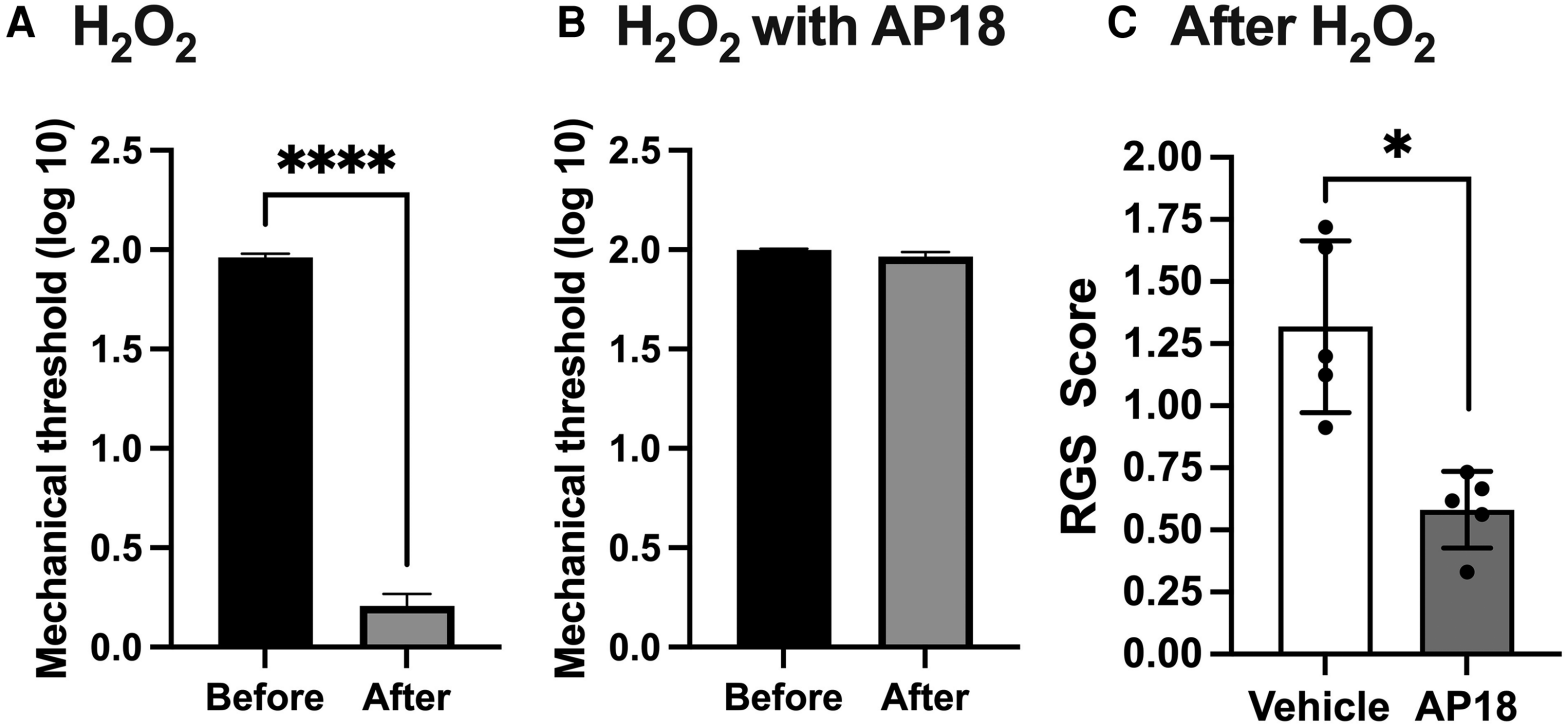
Intraganglionic ROS elicits mechanical hyperalgesia and spontaneous pain via TRPA1. (**A**) H_2_O_2_ (30 *μ*M in 5 *μ*l) was administered directly into TG (intraganlionic) and mechanical sensitivity was assessed 40 min after. Masseter sensitivity was attenuated significantly in rats that received H_2_O_2_ (****p < 0.0001). (**B**). AP18 (20 mM in 5 *μ*l) was administered directly into TG (intraganlionic) and masseter sensitivity was evaluated after H_2_O_2_ administration into TG. (**C**) AP18 (20 mM in 5 *μ*l) or vehicle (1% DMSO, 10% Tween80 in PBS) was co-administered directly into TG (intraganlionic) with H_2_O_2_ (20 *μ*M in 5 *μ*l) and the RGS assay was evaluated (There were no noticeable face grimace behaviors prior to H_2_O_2_ administration). RGS was attenuated significantly in rats that received H_2_O_2_ and AP18 co-administration (*p < 0.005). Data are presented as the mean ± SEM.

Since both PBN and AP18 administered directly into TG effectively attenuated inflammatory hyperalgesia, and ROS are known to directly activate TRPA1, we investigated whether exogenous administration of ROS can induce pain-related responses without peripheral inflammation and whether that effect is mediated by TRPA1. Since the effects of H_2_O_2_ are expected to be short-lived, we evaluated evoked and spontaneous pain responses within an hour of H_2_O_2_ administration. Initially, we directly administered H_2_O_2_ into the trigeminal ganglion (TG) with or without AP18 and assessed masseter mechanical hypersensitivity. Direct administration of H_2_O_2_ into TG (20 *μ*M in 5 *μ*l) resulted in a significant decrease in the mechanical sensitivity of the masseter muscle, indicating the development of profound hyperalgesia (Figure 4A; *p* < 0.0001 for before vs. after). However, when the same concentration of H_2_O_2_ was co-administered with AP18, it effectively prevented H_2_O_2_-induced masseter hyperalgesia (Figure 4B). We also assessed RGS since H_2_O_2_ administration in TG could produce a widespread spontaneous pain not limited to selective orofacial regions. Animals administered with either AP18 or vehicle did not show any changes in facial expression prior to H_2_O_2_ administration. However, when H_2_O_2_ was administered, rats exhibited visible changes in facial expressions, indicated by a high RGS score. The increased RGS score due to H_2_O_2_ treatment was significantly attenuated by co-administration with AP18 (Figure 4C, *p* < 0.005 for AP18 vs. vehicle groups).

### 3.5. ROS induces TRPA1 upregulation in TG

**Figure 5:**
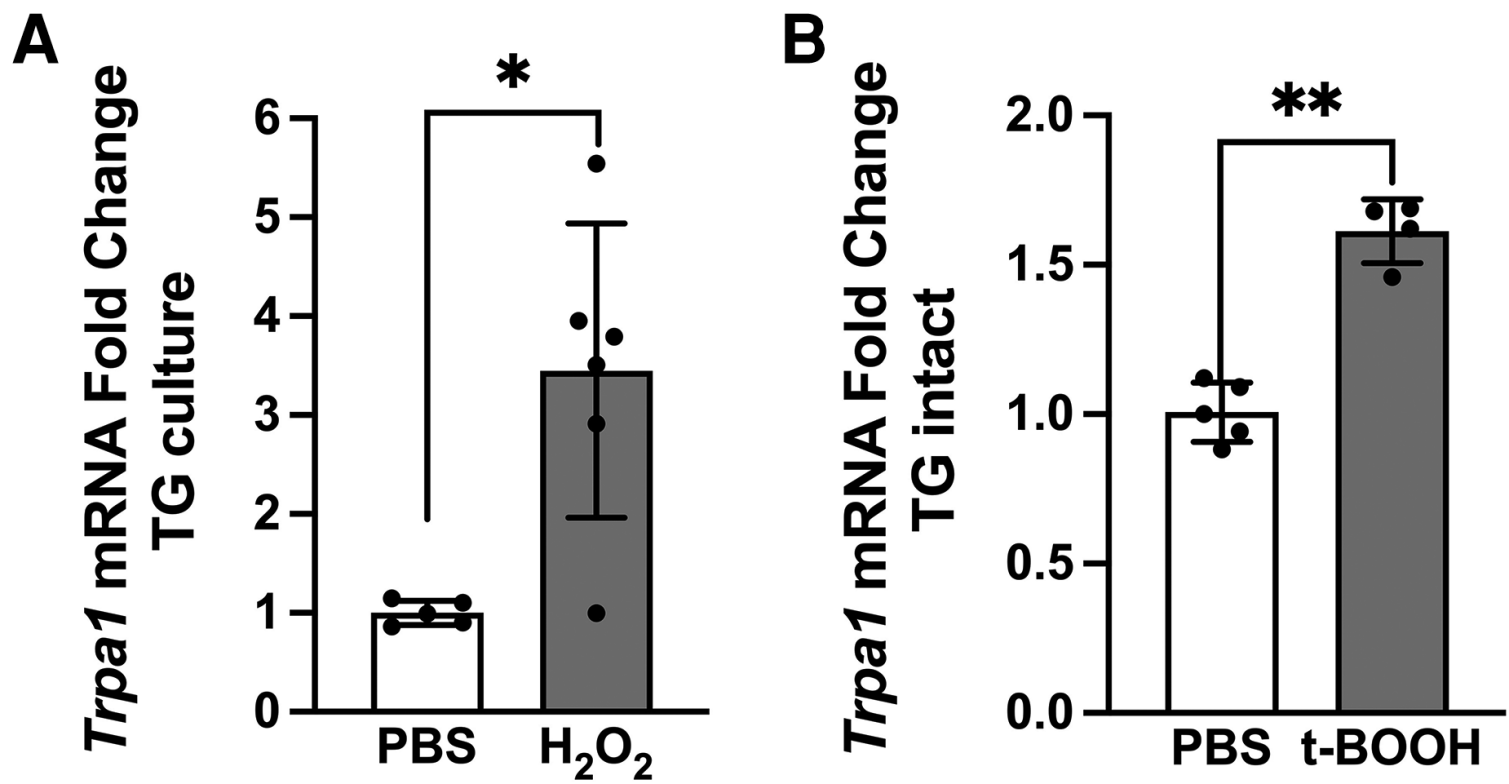
Intraganglionic ROS mediates TRPA1 upregulation. (**A**) H_2_O_2_ (10 *μ*mol, 1 h) treatment in TG culture induces significant increase in *Trpa1* mRNA expression (*p < 0.005). (**B**) Four rats implanted with cannula in left TG received the ROS donor t-BOOH (2 *μ*mol in 10 *μ*l, intragangionic) for two consecutive days. TG were extracted 24 h after the last injection for RT-PCR. Each sample was analyzed in triplicates. t-BOOH treatment in intact TG induces significant increase in *Trpa1* mRNA expression (**p < 0.0001). Data are presented as the mean ± SEM.

**Figure 6:**
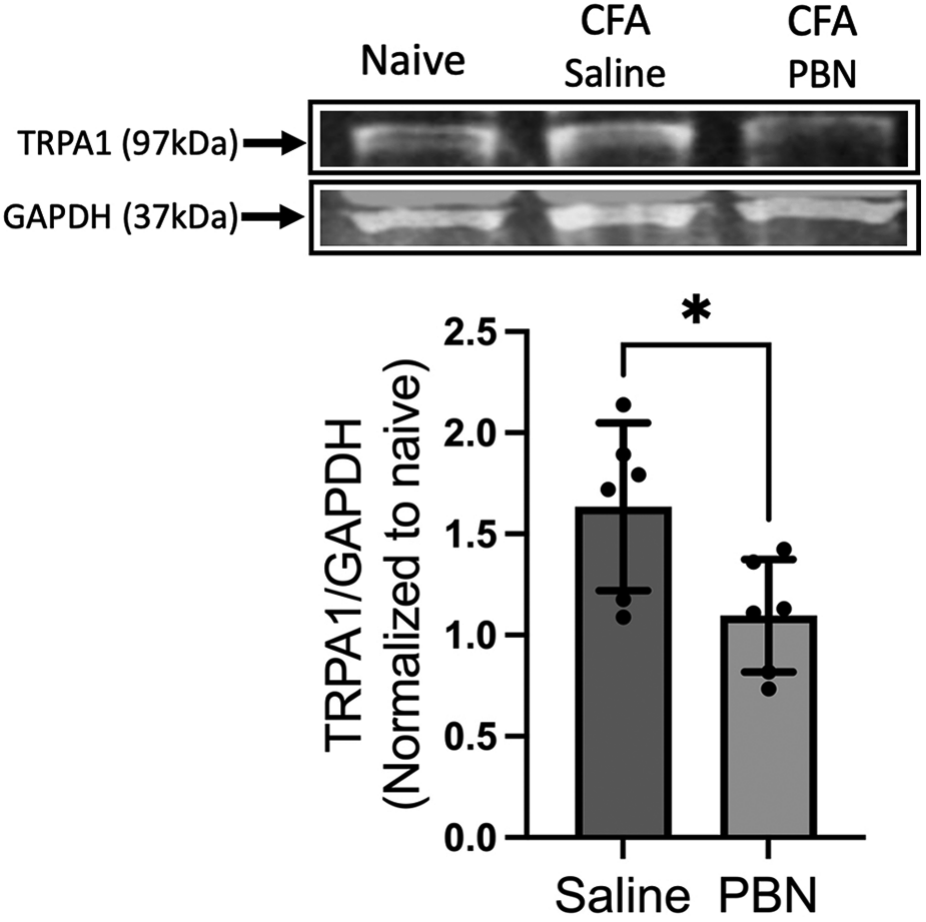
Effects of intraganglionic PBN treatment on CFA-induced upregulation of TRPA1 protein expression. (Top) Representative blots for TRPA1 protein in TG of naïve rats, CFA-inflamed rats (7 days post CFA) with either vehicle or (PBN 0.1 mg in 5 *μ*l) administration. Vehicle or PBN was administered directly into TG (intraganlionic) on days 1, 3, and 7 after the CFA injection in the masseter muscle. (Bottom) Averaged relative optical density (TRPA1/GAPDH). (*p < 0.05). Data are presented as the mean ± SEM.

Since intraganglionic ROS have direct access to transcriptional machineries within the soma, we examined whether ROS are involved in the transcription of the *Trpa1* gene in TG. Relative changes in the mRNA levels of the *Trpa1* gene was determined from H_2_O_2_-treated primary TG culture samples (10 *μ*mol, 1 h). H_2_O_2_ treatment induced a significant increase in TRPA1 (Figure 5A, *p *< 0.05 for H_2_O_2_ vs. naive groups). To confirm that ROS build up in TG can induce a transcriptional increase of *Trpa1*, we administered a ROS donor, t-BOOH, directly into TG of intact animals without inflammation. t-BOOH significantly upregulated the transcript levels of TRPA1 in TG (Figure 5B, *p* < 0.0001 for t-BOOH vs. naive groups), suggesting that ROS alone is sufficient to induce transcriptional changes of *Trpa1* in TG. In our previous study, we showed that CFA-induced masseter inflammation results in a time-dependent increase in the level of *Trpa1* mRNA in TG (33). The level of *Trpa1* mRNA TG is significantly upregulated on days 1, 3 and 7 compared to the baseline. To examine whether ROS buildup in TG can increase TRPA1 protein expression, we administered PBN or vehicle directly into TG on days 1, 3, and 7 of CFA treatment. Here, we confirmed that TRPA1 protein expression is significantly upregulated on day 7 following CFA treatment in the masseter muscle in vehicle treated rats compared to that of naïve rats (Figure 6). However, TRPA1 expression levels in PBN treated rats were significantly lower compared to vehicle treated rats (Figure 6, *p *< 0.05 for Saline vs. PBN groups), suggesting that excess intraganglionic ROS under inflammatory conditions induce upregulation of TRPA1 expression in TG.

## 4. Discussion

We have previously shown that masseter muscle inflammation by CFA results in a significantly elevated level of ROS within the TG of intact animals 24 h after the treatment (20). However, the extent to which ROS expression is altered within TG under pathological pain conditions, as well as how ROS levels in TG contribute to pain and hyperalgesia, is unknown. In the current study, CFA-induced ROS accumulation in TG is maintained at least for 14 days. Such prolonged elevation of ROS in TG could cause a sustained increase in pronociceptive gene expression (40), generation of reactive aldehydes via ROS-dependent lipid peroxidation which could further amplify oxidative stress (2), and activation of TRPA1 by reactive aldehydes that exacerbate neurogenic inflammation (13, 41).

Several findings of the current study provide strong evidence that ROS confined to TG are sufficient to functionally contribute to inflammatory pain responses. First, the time course of ROS elevation paralleled the period of profound mechanical hyperalgesia at the inflamed site and, second, that intraganlionic PBN effectively attenuated CFA-induced mechanical hyperalgesia during this period. It is interesting to note that repetitive treatment with PBN had a cumulative analgesic effect on inflammatory mechanical hyperalgesia. Since PBN has a terminal half-life of 2.01 +/- 0.35 h when administered intravenously in Sprague Dawley rats (42), it is unlikely that the cumulative analgesic effect is due to PBN accumulation in TG. Instead, scavenging ROS could prevent subsequent generation of reactive aldehydes that exert prolonged activity, and therefore act as a second toxic messenger augmenting initial ROS events (28). Thus, ROS-initiated downstream signaling cascades could maintain pain hypersensitivity even after the excess ROS level dissipates to that of the pre-CFA level. Additionally, preemptive treatment of TG with PBN transiently, but significantly, attenuated CFA-induced mechanical hyperalgesia, suggesting that intraganlionic ROS also play a role in the development of inflammatory hyperalgesia. Preemptive administration of ROS scavengers effectively ameliorate inflammatory, neuropathic, and chemotherapy-induced pain responses when given systemically (7, 8, 18, 31, 43). While findings from these studies demonstrated the importance of ROS in local tissue, as well as in the spinal cord dorsal horn, in the development of pathological pain responses, a relative contribution of intraganlionic ROS has not been systematically examined. Lastly, our data showed that direct activation of ROS cascades in TG without peripheral inflammation is sufficient to elicit spontaneous pain. While we did not examine persistent effects of H_2_O_2_ on spontaneous pain, our data are consistent with a recent study that showed a rapid and persistent mechanical hypersensitivity following direct administration of H_2_O_2_ into the DRG of naïve rats (44). Together, our data suggest that TG is an important source of ROS and that intraganlionic ROS participate in the pathogenesis of inflammatory pain, making TG a viable therapeutic target for orofacial pain management.

There is ample evidence that ROS mediate their cellular effects via multiple types of TRP channels, including TRPA1, TRPV1, and TRPM2. Among those TRP channels, TRPA1 has the highest oxidation sensitivity and is therefore regarded as a redox-sensitive channel (11, 45). ROS directly activate TRPA1 by inducing cysteine oxidation and by promoting disulfide formation between proximal cysteine residues in TRPA1 (46). Direct application of H_2_O_2_ to meninges, for example, induces electrical spiking activity of trigeminal nerves in a TRPA1-dependent manner (47). ROS can also enhance the spontaneous release of glutamate from presynaptic terminals onto spinal cord dorsal horn neurons through TRPA1, inducing central sensitization at the central terminals of primary afferent neurons (19). ROS can also indirectly activate TRPA1 by the nonenzymatic generation of endogenous reactive aldehydes (48). There is strong evidence that oxidative aldehydes, such as 4-hydroxy-2E-nonenal (4-HNE), activate TRPA1 through covalent modification of cysteine and lysine residues located within the amino-terminal cytoplasmic domain of the channel (13, 49, 50), and that 4-HNE activation of TRPA1 mediates inflammatory and neuropathic pain (12, 13). Furthermore, TRPA1 activation by H_2_O_2_ has been shown to evoke additional H_2_O_2_ release in melanoma cell lines that further amplify the oxidative stress (51). Therefore, ROS and TRPA1 interactions in any stage of nociceptive processing can lead to sensitization of neurons, promoting pain hypersensitivity. Here, for the first time, we characterize ROS effect of TRPA1 expression within TG and investigate the functional relationship between intraganlionic ROS and TRPA1.

In the current study, we demonstrate that the blockade of intraganglionic TRPA1 effectively mitigated the CFA-induced mechanical hyperalgesia at various time points during the inflammation. It is possible that excess ROS within TG can directly and indirectly activate TRPA1 expressed in soma of masseter afferents (34), contributing to inflammatory muscular hyperalgesia. Our data demonstrating that spontaneous pain and masseter hypersensitivity elicited by exogenous administration of H_2_O_2_ into TG is blocked by a TRPA1 antagonist supports this hypothesis. Our hypothesis is further supported by a recent study showing that TRPA1 activation by ROS and carbonyl species in the soma of TG maintain glyceryl trinitrate-induced periorbital allodynia (41). Collectively, these observations provide strong evidence that interactions between ROS and TRPA1 within sensory ganglia functionally contribute to chronic pain. Previous studies have demonstrated that the soma of primary afferent neurons functionally contribute to chronic pain by intraganlionic communication between a population of DRG neurons, also referred to as cross-excitation (52, 53). More recent studies implicate intraganlionic sensory transmission as a mechanism by which sensory input from one type of tissue can exert sensitizing effects through widespread actions of chemical mediators, such as calcitonin gene-related peptide, on neighboring neurons within the ganglia (54, 55). In this context, cellular mechanisms mediating intraganlionic ROS-TRPA1 interactions, as well as the relative contribution of such interactions in various chronic pain conditions, merit further investigation and could potentially be a therapeutic target.

TRPA1 expression level is highly correlated with inflammatory hyperalgesia (24, 56, 57). We previously reported that CFA-induced masseter hyperalgesia is accompanied by significant up-regulation of *Trpa1* mRNA expression in TG (33, 58). While our understanding of the mechanisms regulating *Trpa1* transcription in TG under inflammatory conditions is limited, our data suggest that intraganlionic ROS can activate the transcriptional machineries of the *Trpa1* gene. ROS could lead to *Trpa1* gene upregulation by inflammatory cytokines. TNF-α and IL-1β increase TRPA1 expression in human lung epithelial cells (59), and TRPA1 expression was also significantly upregulated by TNF-α in human odontoblast-like cells (60). Notably, mice with a null mutation of glycoprotein 130, a signal transducing subunit for IL-6, show reduced *Trpa1* mRNA expression in DRG (25). It is well known that oxygen-derived free radicals, such as H_2_O_2,_ stimulate the synthesis of multiple inflammatory cytokines and chemokines in a variety of cells. H_2_O_2_ increases the production of IL-6 and IL-8 in airway epithelia (61), IL-1*β*, IL-6, TNF-*α*, and TGF-*β*1 in cardiac fibroblasts (62), TNF-α in DRG cells (10), and IL-6 in TG neurons and satellite glia (20). Interactions of ROS with NF*κ*B is also well known (63). In a previous study, we demonstrated that the accumulation of ROS in the TG upregulated the mRNA and protein levels of IL-6 and chemokine (C-X-C motif) ligand 2 (CXCL2) via the transient receptor potential melastatin 2 (TRPM2) channels, which is expressed in both neurons and satellite glial cells (20). It is known that TRPM2 is directly activated by ROS, including H_2_O_2_ (64). Therefore, the accumulation of ROS in TG can activate TRPM2, which in turn generates inflammatory cytokines and chemokines via NF*κ*B pathways, leading to increased expression of the TRPA1 channel.

Oxidative stress is also known to cause a wide range of DNA modifications, such as base modifications, strand breakage, and chromosomal rearrangements. Such modifications have been shown to interfere with the activities of DNA methyl transferases (DNMTs), resulting in global hypomethylation (65). As a prototypical epigenetic factor, DNA methylation plays an important role in regulating gene expression, and alterations in DNA methylation in DRG play a critical role in the underlying mechanisms of many types of somatic chronic pain conditions (66, 67, 68). CFA-induced masseter inflammation leads to both global reductions in DNA methylation and to downregulation of DNMT1 and DNMT3a expression in TG, and the reduction of DNMT3a leads to a decrease in promoter methylation of *Trpv1* and *Trpa1*, which results in their upregulation (37). Thus, intraganglionic ROS could serve as the mechanistic link between peripheral inflammation and the transcription of pro-nociceptive genes, such as *Trpa1*, by reducing DNA methylation.

Taken together, results from this study demonstrate that excess intraganlionic ROS generated in response to peripheral inflammation functionally contributes to pain and hyperalgesia. Thus, the soma of nociceptors, in addition to peripheral and central terminals, are important sites for the pathogenesis of inflammatory pain. Furthermore, oxidative stress induces transcriptional upregulation of pronociceptive genes, ultimately contributing to peripheral sensitization. Therefore, any conditions that exacerbate ROS accumulation within somatic sensory ganglia can aggravate pain responses. Recent studies have shown that natural antioxidants such as curcumin (69), resveratrol (70), Vitamin E (71), and catechins in green tea (72) reduce pain and inflammation in animal models of pain. These treatments have the potential to reduce ganglionic ROS and help alleviate inflammatory pain.

## Data Availability

The original contributions presented in the study are included in the article, further inquiries can be directed to the corresponding author.
